# Diophantine equations in separated variables

**DOI:** 10.1007/s10998-017-0195-y

**Published:** 2017-08-01

**Authors:** Dijana Kreso, Robert F. Tichy

**Affiliations:** Graz University of Technology, Steyrergasse 30/II, 8010 Graz, Austria

**Keywords:** Diophantine equations, Monodromy group, Permutation groups, Polynomial decomposition

## Abstract

We study Diophantine equations of type $f(x)=g(y)$, where both *f* and *g* have at least two distinct critical points (roots of the derivative) and equal critical values at at most two distinct critical points. Various classical families of polynomials $(f_n)_n$ are such that $f_n$ satisfies these assumptions for all *n*. Our results cover and generalize several results in the literature on the finiteness of integral solutions to such equations. In doing so, we analyse the properties of the monodromy groups of such polynomials. We show that if *f* has coefficients in a field *K* of characteristic zero, and at least two distinct critical points and all distinct critical values, then the monodromy group of *f* is a doubly transitive permutation group. In particular, *f* cannot be represented as a composition of lower degree polynomials. Several authors have studied monodromy groups of polynomials with some similar properties. We further show that if *f* has at least two distinct critical points and equal critical values at at most two of them, and if $f(x)=g(h(x))$ with $g, h\in K[x]$ and $\deg g>1$, then either $\deg h\le 2$, or *f* is of special type. In the latter case, in particular, *f* has no three simple critical points, nor five distinct critical points.

## Introduction

Diophantine equations of type $f(x)=g(y)$ have been of long-standing interest to number theorists. A defining equation of an elliptic curve is a prominent example of such equations. By Siegel’s classical theorem, it follows that an irreducible algebraic curve defined over a number field has only finitely many *S*-integral points, unless it has genus zero and no more than two points at infinity. Ever since Siegel’s theorem, one of the driving questions was to classify polynomials *f*, *g* for which the equation $f(x)=g(y)$ has infinitely many solutions in *S*-integers *x*, *y*. The classification was completed by Bilu and Tichy [5] in 2000, by building on the work of Fried and Schinzel. It turns out that such *f* and *g* must be representable as a composition of lower degree polynomials in a certain prescribed way.

The possible ways of writing a polynomial as a composition of lower degree polynomials were studied by several authors, starting with Ritt in the 1920’s in his classical paper [25]. Ritt’s and related results have applications to Diophantine equations, but also to various other areas of mathematics, such as complex analysis, arithmetic dynamics, finite geometries, etc. See e.g. [26] and [36] for an overview of the theory and applications.

The theorem of Bilu and Tichy was used to prove the finiteness of integral solutions to various equations of type $f(x)=g(y)$, where $f, g\in \mathbb {Q}[x]$. For more details, see our recent survey paper [18]. In this paper, we utilize some standard methods of Galois theory for maps between curves, to give simple and unifying proof of most of those results.

For a number field *K*, a finite set *S* of places of *K* that contains all Archimedean places and the ring $\mathcal {O}_S$ of *S*-integers of *K*, we say that the equation $f(x)=g(y)$ has infinitely many solutions *x*, *y* with a bounded $\mathcal {O}_S$-denominator if there exists a nonzero $\delta \in \mathcal {O}_S$ such that there are infinitely many solutions $x, y\in K$ with $\delta x, \delta y\in \mathcal {O}_S$.

For a polynomial *f*, the roots of the derivative $f'$ are called *critical points*, and the values of *f* at critical points are called *critical values*. If for critical points $\beta _i$’s of *f*, one has $f(\beta _i)\ne f(\beta _j)$ when $\beta _i\ne \beta _j$, then *f* is said to have *all distinct critical values*.

### Theorem 1.1

Let *K* be a number field, *S* a finite set of places of *K* that contains all Archimedean places, $\mathcal {O}_S$ the ring of *S*-integers of *K*, and $f,g\in K[x]$ with $\deg f\ge 3, \deg g\ge 3$. If *f* and *g* both have at least two distinct critical points and all distinct critical values, then the equation $f(x)=g(y)$ has infinitely many solutions *x*, *y* with a bounded $\mathcal {O}_S$-denominator if and only if $f(x)=g(\mu (x))$ for some linear $\mu \in K[x]$.

### Corollary 1.2

Let *K* be a number field, *S* a finite set of places of *K* that contains all Archimedean places and $\mathcal {O}_S$ the ring of *S*-integers of *K*. Let $a_1, a_2, a_3, b_1, b_2\in K$ with $a_1 a_2b_1b_2\ne 0$. Let further $n_1, n_2, m_1, m_2\in \mathbb {N}$ be such that $n_1>n_2, m_1>m_2, \gcd (n_1, n_2)=1, \gcd (m_1, m_2)=1$ and $n_1, m_1\ge 3$. Then the equation

1.1 $$\begin{aligned} a_1x^{n_1}+a_2x^{n_2}+a_3=b_1y^{m_1}+b_2y^{m_2} \end{aligned}$$

has infinitely many solutions *x*, *y* with a bounded $\mathcal {O}_S$-denominator if and only if for some linear $\mu \in K[x]$ we have

1.2 $$\begin{aligned} a_1x^{n_1}+a_2x^{n_2}+a_3=(b_1x^{m_1}+b_2x^{m_2})\circ \mu (x). \end{aligned}$$

Corollary 1.2 follows from Theorem 1.1. Namely, if $f(x)=a_1x^{n_1}+a_2x^{n_2}+a_3$, then clearly $f'(x)=x^{n_2-1}\left( a_1n_1x^{n_1-n_2}+a_2n_2\right)$, so $f'$ has at least two distinct critical points. Also, $xf'(x)=n_1(f(x)-a_3) - a_2(n_1-n_2)x^{n_2}$. If $f(\alpha )= f(\beta )$ for distinct critical points $\alpha$ and $\beta$ of *f*, then $\alpha ^{n_2}=\beta ^{n_2}$. Then from $f(\alpha )=f(\beta )$ it follows that $\alpha ^{n_1}=\beta ^{n_1}$. Since $\gcd (n_1, n_2)=1$, we have $\alpha =\beta$, a contradiction. If (1.2) holds, then one can show that either $\mu (0)=0$, or $n_1=m_1\le 3$. Details can be found in [15], where equations of type (1.1) with one or both trinomials replaced by polynomials with an arbitrary but fixed number of nonconstant terms, are studied. Corollary 1.2 generalizes the main result of Péter, Pintér and Schinzel [24, Thm. 1], who proved it in the case when $K=\mathbb {Q}$ and $\mathcal {O}_S=\mathbb {Z}$. They generalized the results of Mignotte and Pethő [22, Thm. 1], of Bugeaud and Luca [6, Thm 6.2], and of Luca [21, Prop. 3]. Polynomials with a fixed number of nonconstant terms are called lacunary. Such polynomials have been studied from various viewpoints. We remark that in the study of Diophantine equations of type $f(x)=g(y)$ where *f* and *g* are lacunary polynomials, of importance are results about the behavior of lacunary polynomials with respect to functional composition. The latter topic has been of interest already to Erdős and Renyi in the 1940’s. Some remarkable results on this topic have been achieved in the last decade. For more details, we direct the reader to [26, 34, 35].

### Corollary 1.3

Let $m> n\ge 3$ be integers. The equation $x^{n}+x^{n-1}+\cdots +x+1=y^{m}+y^{m-1}+\cdots +y+1$ has only finitely many rational solutions *x*, *y* with a bounded denominator.

Let $f(x)=x^{n}+x^{n-1}+\cdots +x+1$. Then $f(x)+(x-1)f'(x)=(n+1)x^{n}$, and hence $xf'(x)+(x-1)f''(x)=(n+1)nx^{n-1}$. Clearly, *f* has only simple critical points. Furthermore, *f* has all distinct critical values unless there exist two distinct critical points $\alpha$ and $\beta$ of *f* such that $\alpha ^n=\beta ^{n}$. Assume that $\alpha ^n=\beta ^{n}$. Since $f(\alpha )=f(\beta )$, it also follows that $\alpha ^{n-1}+\cdots +\alpha +1=\beta ^{n-1}+\cdots +\beta +1$. Note that $\alpha , \beta \ne 1$ since $f'(1)=n+(n-1)+\cdots +2+1>0$. Thus, $(1-\alpha ^n)/(1-\alpha )=(1-\beta ^n)/(1-\beta )$ and hence $\alpha =\beta$, a contradiction. Therefore, Corollary 1.3 follows from Theorem 1.1. In particular, there are only finitely many integer solutions to this equation. The latter result was shown by Davenport, Lewis and Schinzel [8].

Further corollaries of Theorem 1.1 are given in Sect. 5. In the sequel we present our methods.

For a field *K* and $f\in K[x]$ with $f'(x)\ne 0$, the Galois group of $f(x)-t$ over *K*(*t*), where *t* is transcendental over *K*, seen as a permutation group of the roots of this polynomial, is called the *monodromy group* of *f*, and is denoted by ${{\mathrm{Mon}}}(f)$. A lot of information about the polynomial *f* is encoded into its monodromy group. To the proof of Theorem 1.1, in Sect. 3 we show that if *K* is a field of characteristic zero and $f\in K[x]$ has at least two distinct critical points and all distinct critical values, then ${{\mathrm{Mon}}}(f)$ is a doubly transitive permutation group. In particular, such *f* cannot be represented as a composition of lower degree polynomials. Polynomials with only simple critical points and all distinct critical values are called Morse by Serre [27]. He showed that for an arbitrary field *K* and Morse $f\in K[x]$ such that ${{\mathrm{char}}}(K)\not \mid \deg f$, the monodromy group of *f* is symmetric. Turnwald [32] showed that in Serre’s result the condition on *f* can be relaxed from requiring that it has all simple critical points to requiring that it has one simple critical point (and all distinct critical values). These kind of questions were previously studied by Hilbert, Birch and Swinerton-Dyer, etc. One may find more details in Turnwald’s paper. In Sect. 3, we recover these results. In [16], it is shown that two Morse polynomials with rational coefficients, of distinct degrees which are both $\ge 3$, cannot have infinitely many equal values at integer points. This result, generalized by Theorem 1.1, does not imply Corollary 1.2, nor the aforementioned results in [6, 21, 24].

We say that polynomial *f* has *equal critical values at at most two distinct critical points* if there do not exist three distinct critical points $x_0, y_0, z_0$ of *f* such that $f(x_0)=f(y_0)=f(z_0)$. Some well-known families of polynomials $(f_n)_n$ satisfy that $f_n$ for all *n* has equal critical values at at most two distinct critical points.

For example, it is shown in [2] that this holds when $f_n(x)=x(x+1)\cdots (x+n-1)$. There are many results in the literature about Diophantine equations of type $f(x)=g(y)$, where $f(x)=x(x+1)\cdots (x+n-1)$, see e.g. [2, 3, 11]. For instance, by the celebrated theorem of Erdős and Selfridge [11], the equation $x(x+1)\cdots (x+n-1)=y^n$ for $m, n\ge 2$ has no solutions in positive integers *x*, *y*.

### Proposition 1.4

Let *K* be a field with ${{\mathrm{char}}}(K)=0$ and let $f\in K[x]$ have at least two distinct critical points and equal critical values at at most two distinct critical points. If $f(x)=g(h(x))$, where $g, h\in K[x], t=\deg g>1$ and $k=\deg h> 2$, then the derivative of *f* satisfies either

1.3 $$\begin{aligned} f'(x)=a'(x-x_0)^{k_0t-1}(x-y_0)^{l_0t-1}(kx -k_0y_0-l_0x_0), \end{aligned}$$

for some $a'\in K, k_0, l_0\ge 1$ such that $k_0+l_0=k$ and distinct $x_0,y_0\in \overline{K}$, or

1.4 $$\begin{aligned} f'(x)=a' (x-x_0)^{2t_0+1}(x-y_0)^{t_0}(x-x_1)^{2t_1+1}(x-y_1)^{t_1}, \end{aligned}$$

for some $a'\in K, t_0, t_1\ge 1$ such that $t_0+t_1=t-1$, and distinct $x_0, x_1, y_0, y_1\in \overline{K}$ that satisfy $3x_0=x_1+2y_1$ and $3x_1=x_0+2y_0$.

If $f(x)=g(h(x))$, where $g, h\in K[x], t=\deg g>1$ and $k=\deg h=2$, then either *g* has no two distinct critical points, in which case (1.3) holds, or ${{\mathrm{Mon}}}(g)$ is doubly transitive.

Finally, if *f* is indecomposable and $\deg f\ge 6$, then ${{\mathrm{Mon}}}(f)$ is doubly transitive.

By Proposition 1.4 it follows that if *K* is a field with ${{\mathrm{char}}}(K)=0, f\in K[x]$ has at least three simple critical points, equal critical values at at most two distinct critical points, and $f(x)=g(h(x))$, where $g, h\in K[x]$ and $\deg g>1$, then $\deg h\le 2$. Namely, by Proposition 1.4, if this is not the case, then either (1.3) or (1.4) holds. If (1.3) holds, then $k_0t=l_0t=2$, and thus $t=2$ (since $t>1$ by assumption), $k_0=l_0=1$ and $\deg h=k=2$, a contradiction. If (1.4) holds, then $t_0=t_1=1$ and either $2t_0+1=1$ or $2t_1+1=1$, a contradiction.

It is easy to see (see Lemma 3.7 and the text below) that if *f* has only simple critical points and equal critical values at at most two distinct critical points, and $f(x)=g(h(x))$, where $g, h\in K[x]$ and $\deg g>1$, then $\deg h\le 2$. This fact was used in [2, 9, 10, 28] in the study of Diophantine equations of type $f(x)=g(y)$ via Bilu and Tichy’s theorem, to find the possible decompositions of *f* and *g*. The proofs in those papers are completed by a lengthy analysis of subcases implied by the criterion and depend on the comparison of coefficients of the involved polynomials. Results of these papers are, to the most part, generalized by the following theorem. In what follows, by saying that the derivative of *g* satisfies (1.3) or (1.4), we mean that (1.3) or (1.4) hold with *f* replaced by *g*.

### Theorem 1.5

Let *K* be a number field, *S* a finite set of places of *K* that contains all Archimedean places, $\mathcal {O}_S$ the ring of *S*-integers of *K* and $f,g\in K[x]$ such that $\deg f\ge 3, \deg g\ge 3$ and $\deg f< \deg g$.

If both *f* and *g* have at least two distinct critical points and equal critical values at at most two distinct critical points, and their derivatives do not satisfy (1.3) nor (1.4), then the equation $f(x)=g(y)$ has only finitely many solutions with a bounded $\mathcal {O}_S$-denominator, unless either $(\deg f, \deg g)=(3, 5)$, or *f* is indecomposable and $g(x)=f(\nu (x))$ for some quadratic $\nu \in K[x]$.

If *f* and *g* have only simple critical points and equal critical values at at most two distinct critical points, then the equation $f(x)=g(y)$ has only finitely many solutions with a bounded $\mathcal {O}_S$-denominator, unless either $(\deg f, \deg g)\in \{(3, 4), (3, 5), (4, 5)\}$, or *f* is indecomposable and $g(x)=f(\nu (x))$ for some quadratic $\nu \in K[x]$.

Theorem 1.5 is proved in Sect. 4. In relation to Theorem 1.5, we further list all pairs of polynomials (*f*, *g*) with $(\deg f, \deg g)\in \{(3, 4), (3, 5), (4, 5)\}$ that satisfy the assumptions of the theorem, and are such that the equation $f(x)=g(y)$ has infinitely many solutions with a bounded $\mathcal {O}_S$-denominator, see Theorem 4.2. It is easy to check if $g(x)=f(\nu (x))$ holds for some polynomial $\nu$ with $\deg \nu =2$, e.g. by comparison of coefficients of the involved polynomials. Note that if this holds, then there are infinitely many rational solutions *x*, *y* with a bounded $\mathcal {O}_S$-denominator of the equation $f(x)=g(y)$, namely $(x, y)=(\nu (t), t)$, where $t\in \mathcal {O}_S$. In particular, note that the equation $a_1x^{n_1}+a_2x^{n_2}=a_1y^{2n_1}+a_2y^{2n_2}$, where $a_1, a_2$ are nonzero elements of a number field, $n_1, n_2\in \mathbb {N}, n_1\ge 3$, and $\gcd (n_1, n_2)=1$, satisfies the assumptions of the theorem and has infinitely many solutions $(x, y)=(t^2, t)$, where $t\in \mathcal {O}_S$. In Sect. 4, we discuss the necessity of the assumption $\deg f\ne \deg g$ in Theorem 1.5. In Sect. 5, we list various corollaries of this theorem.

We remark that Theorem 1.1 and Theorem 1.5 are ineffective since they rely on the main result of [5], which in turn relies on Siegel’s classical theorem on integral points on curves, which is ineffective.

## The finiteness criterion

In this section we present the finiteness criterion of Bilu and Tichy [5].

Let *K* be a field of characteristic zero, $a, b\in K {\setminus }\{0\}, m, n\in \mathbb {N}, r\in \mathbb {N}\cup \{0\}, p \in K[x]$ be a nonzero polynomial (which may be constant) and $D_{n} (x,a)$ be the *n*-th Dickson polynomial with parameter *a* given by

2.1 $$\begin{aligned} D_n(x,a)=\sum _{j=0}^{\lfloor n/2 \rfloor } \frac{n}{n-j} {n-j \atopwithdelims ()j} (-a)^{j} x^{n-2j}. \end{aligned}$$

It is well known that

2.2 $$\begin{aligned} D_k(x,a)=2\sqrt{a}^k T_k(x/2\sqrt{a}), \end{aligned}$$

where $T_k(x)=\cos (k \arccos x)$ is the *k*th Chebyshev polynomial of the first kind. For various properties of Dickson polynomials, see [5, Sec. 3]. Some will be recalled in Sect. 3.1.

*Standard* pairs of polynomials over *K* are listed in the following table.

**Table:** 

Kind	Standard pair (or switched)	Parameter restrictions
First	$(x^m, a x^rp(x)^m)$	$r<m, \gcd (r, m)=1,\ r+ \deg p > 0$
Second	$(x^2,\left( a x^2+b)p(x)^2\right)$	–
Third	$\left( D_m(x, a^n), D_n(x, a^m)\right)$	$\gcd (m, n)=1$
Fourth	$(a ^{\frac{-m}{2}}D_m(x, a), -b^{\frac{-n}{2}}D_n (x,b))$	$\gcd (m, n)=2$
Fifth	$\left( (ax^2 -1)^3, 3x^4-4x^3\right)$	–

We further call the pair

$$\begin{aligned} \left( D_m \left( x, a^{n/d}\right) , - D_n \left( x \cos (\pi /d), a^{m/d}\right) \right) \ \text {(or switched)}, \end{aligned}$$

with $d=\gcd (m, n)\ge 3$ and $\cos (2\pi /d)\in K$, a *specific pair* over *K*. One easily sees that if $b, \cos (2\alpha )\in K$, then $D_n(x\cos \alpha , b)\in K[x]$.

### Theorem 2.1

Let *K* be a number field, *S* a finite set of places of *K* that contains all Archimedean places, $\mathcal {O}_S$ the ring of *S*-integers of *K*, and $f, g\in K[x]$ nonconstant. Then the following assertions are equivalent.

- The equation $f(x)=g(y)$ has infinitely many solutions with a bounded $\mathcal {O}_S$-denominator;
- We have
  2.3 $$\begin{aligned} f(x)=\phi \left( f_{1}\left( \lambda (x)\right) \right) \quad {\hbox {and}} \quad g(x)=\phi \left( g_{1}\left( \mu (x)\right) \right) , \end{aligned}$$
  where $\phi \in K[x], \lambda , \mu \in K[x]$ are linear polynomials, and $\left( f_{1},g_{1}\right)$ is a standard or specific pair over *K* such that the equation $f_1(x)=g_1(y)$ has infinitely many solutions with a bounded $\mathcal {O}_S$-denominator.

We remark that in [4], in relation to Theorem 2.1, the authors asked and answered the following question: *Given* $f, g\in \mathbb {Q}[x]$, *is it true that all but finitely many rational solutions with a bounded denominator of the equation* $f(x) = g(y)$ *also satisfy the equation* $f_1(\lambda (x))=g_1(\mu (y))$? Unfortunately, this is not true in general, and some counterexamples are not hard to find. In [4, Thm. 4], the authors found all counterexamples to this statement.

## Polynomial decomposition via Galois theory

Throughout this section *K* is an arbitrary field with ${{\mathrm{char}}}(K)=0$.

A polynomial $f\in K[x]$ with $\deg f>1$ is called *indecomposable* (over *K*) if it cannot be written as the composition $f(x)=g(h(x))$ with $g,h\in K[x], \deg g>1$ and $\deg h>1$. Otherwise, *f* is said to be *decomposable*. Any representation of *f* as a functional composition of polynomials of degree $>1$ is said to be a *decomposition* of *f*. If $\mu \in K[x]$ is linear, then there exists $\mu ^{\langle -1\rangle }\in K[x]$ such that $(\mu \circ \mu ^{\langle -1\rangle })(x)=(\mu ^{\langle -1\rangle }\circ \mu )(x)=x$. By comparison of degrees one sees that no such polynomial exists when $\deg \mu >1$.

Note that for decomposable $f\in K[x]$ we may write without loss of generality

3.1 $$\begin{aligned} \begin{aligned} f(x)=g(h(x)),\ \text { where }\ g, h\in K[x], \ \deg g\ge 2, \deg h \ge 2,\ h \ \text { is monic and } \ h(0)=0. \end{aligned} \end{aligned}$$

Namely, if $f=g\circ h$ with $g, h\in K[x]{\setminus } K$, then there exists a linear $\mu \in K[x]$ such that $\mu \circ h$ is monic and $\mu (h(0))=0$. Clearly $f=\left( g\circ \mu ^{\langle -1\rangle }\right) \circ \left( \mu \circ h\right)$.

### Proposition 3.1

Let *K* be a field with ${{\mathrm{char}}}(K)=0$. Then *f* is indecomposable over *K* if and only if *f* is indecomposable over $\overline{K}$.

Proposition 3.1 is due to Fried and McRae [13]. To the proof, let $f\in K[x]$ and assume that it is decomposable over $\overline{K}$. Then write $f=g\circ h$, where $g, h\in \overline{K}$ are such that $\deg g \ge 2, \deg h\ge 2, h$ is monic and $h(0)=0$, as in (3.1). Comparison of coefficients, starting from the highest-degree coefficient and proceeding inductively, yields $g, h\in K[x]$.

Recall the definition of the monodromy group of a polynomial from the introduction. By Gauss’s lemma it follows that $f(X)-t$ is irreducible over *K*(*t*), so ${{\mathrm{Mon}}}(f)$ is a transitive permutation group. If ${{\mathrm{char}}}(K)=0$, it follows that $f(X)-t$ is also separable. Let *x* be a root of $f(X)-t$ in its splitting field *L* over *K*(*t*). Then $t=f(x)$ and ${{\mathrm{Mon}}}(f)={{\mathrm{Gal}}}(L/K(f(x)))$ is viewed as a permutation group on the conjugates of *x* over *K*(*f*(*x*)).

Lüroth’s theorem (see [26, p. 13]) states that for fields *K*, *L* satisfying $K\subset L\subseteq K(x)$ we have $L=K(h(x))$ for some $h\in K(x)$. This theorem provides a dictionary between decompositions of $f\in K[x]$ and fields between *K*(*f*(*x*)) and *K*(*x*). Namely, if $f(x)=g(h(x))$, then $K(f(x))\subseteq K(h(x))\subseteq K(x)$. On the other hand, if *L* is a field between *K*(*f*(*x*)) and *K*(*x*), by Lüroth’s theorem it follows that $L=K(h(x))$ for some $h\in K(x)$. Since *f* is a polynomial, *h* can be chosen to be a polynomial by [26, p. 16]. Then $f=g(h(x))$ for some $g\in K[x]$. The fields between *K*(*f*(*x*)) and *K*(*x*) clearly correspond to groups between the two associated Galois groups—${{\mathrm{Gal}}}(L/K(f(x)))={{\mathrm{Mon}}}(f)$ and ${{\mathrm{Gal}}}(L/K(x))$ (the stabilizer of *x* in ${{\mathrm{Mon}}}(f)$).

Find more about the Galois theoretic setup for addressing decomposition questions in [19] and [36]. In [36], this approach, which originated in Ritt’s work [25], is presented in a modernized and simplified language, and various new results are proved. In [19], the authors adopted this modernized language and examined the different ways of writing a cover of curves over a field *K* as a composition of covers of curves over *K* of degree at least 2 which cannot be written as the composition of two lower-degree covers. By the generalization to the framework of covers of curves, which provides a valuable perspective even when one is only interested in questions about polynomials, several improvements on previous work were made possible.

### The monodromy group

We now list some well-known properties of the monodromy group that will be used in the sequel, sometimes without particular reference. In this section as well, *K* is an arbitrary field with ${{\mathrm{char}}}(K)=0$.

First we recall some notions concerning permutation groups. A transitive permutation group *G* acting on a set *X* is called primitive if it preserves no nontrivial partition of *X* (trivial partitions are those consisting either of one set of size $\#X$ or of $\# X$ singletons). A permutation group *G* acting on a set *X* with $\# X\ge 2$ is called doubly transitive when, for any two ordered pairs of distinct elements $(x_1, y_1)$ and $(x_2, y_2)$ in $X^2$, there is $g\in G$ such that $y_1=gx_1$ and $y_2=gx_2$. Every doubly transitive permutation group is primitive. A symmetric group is doubly transitive if it is of degree at least two, and an alternating group is doubly transitive if it is of degree at least four. See [7] for a reminder about transitive group actions.

The following two lemmas are due to Ritt [25] and Fried [12], respectively.

#### Lemma 3.2

If *K* is a field with ${{\mathrm{char}}}(K)=0$ and $f\in K[x]$, then *f* is indecomposable if and only if ${{\mathrm{Mon}}}(f)$ is primitive.

A transitive permutation group is primitive if and only if its point stabilizers are maximal subgroups by [7, Thm. 7.16]. By the above stated Lüroth’s theorem, $f\in K[x]$ is indecomposable if and only if there are no proper intermediate fields of the extension *K*(*x*) / *K*(*f*(*x*)). By the Galois correspondence, this is the same as saying that there are no proper subgroups between ${{\mathrm{Mon}}}(f)$ and its any point stabilizer. This proves Lemma 3.2.

#### Lemma 3.3

If *K* is a field with ${{\mathrm{char}}}(K)=0$ and $f\in K[x]$, then $(f(x)-f(y))/(x-y)$ is irreducible over *K* if and only if ${{\mathrm{Mon}}}(f)$ is doubly transitive.

Let $\phi (x, y)=(f(x)-f(y))/(x-y)\in K[x, y]$. In short, Lemma 3.3 follows from the fact that a permutation group of degree at least three is doubly transitive on *X* if and only if the stabilizer of any $x_0\in X$ acts transitively on $X{\setminus } \{x_0\}$, see [7, Thm. 4.13]. Let *t* be transcendental over *K* and let $x_0$ be any root of $f(x)-t$. Then ${{\mathrm{Mon}}}(f)$ is doubly transitive if and only if $\phi (x, x_0)$ is irreducible over $K(x_0)$. Since $x_0$ and *x* are algebraically independent over *K*, this is equivalent to irreducibility of $\phi (x, y)$ over *K*(*y*), which is by Gauss Lemma equivalent to irreducibility of $\phi (x, y)$ over *K*. For a detailed proof, see [32].

#### Lemma 3.4

If *K* is a field with ${{\mathrm{char}}}(K)=0$ and $e_1,e_2, \ldots , e_k$ are the multiplicities of the roots of $f(x)-x_0$, where $f\in K[x]$ and $x_0\in \overline{K}$, then ${{\mathrm{Mon}}}(f)$ contains an element having cycle lengths $e_1,e_2, \ldots , e_k$. Furthermore, if $n=\deg f$, then ${{\mathrm{Mon}}}(f)$ contains an *n*-cycle.

Lemma 3.4 has been long known in the case $K=\mathbb {C}$, but derived in the language of Riemann surfaces. We refer to Turnwald’s paper [32] for an elementary proof. Proofs of all the above mentioned results can be found in Turnwald’s paper.

A theorem of Schur (see [33, p. 34]) states that *a primitive permutation group of composite degree* *n* *which contains an* *n*-*cycle, is doubly transitive*. Thus, if ${{\mathrm{Mon}}}(f)$ is primitive, then it is doubly transitive as soon as it is of composite degree *n*. Burnside showed (see [33, p. 29]) that *if a transitive permutation group of prime degree is not doubly transitive, it may be identified with a group of affine transformations of* $\mathbb {Z}/p\mathbb {Z}$. The latter two results of Schur and Burnside are classical results about permutation groups and were among the main ingredients of Fried [12] in proving the following theorem.

#### Theorem 3.5

Let *K* be a field with ${{\mathrm{char}}}(K)=0$ and $f\in K[x]$ with $\deg f\ge 3$. The following assertions are equivalent.

i) $(f(x)-f(y))/(x-y)$ is reducible over $\overline{K}$,
ii) either *f*(*x*) is decomposable, or $f(x)=e_1 D_n(c_1x+c_0, \alpha )+e_0$, $n>3$ is a prime and $e_i, c_i,\alpha \in K$, or $f(x)=e_1 D_3(c_1x+c_0,0)+e_0=e_1(c_1x+c_0)^3+e_0$ and $e_i, c_i\in K$, where $D_n(x, a)$ is the Dickson polynomial of degree *n* with parameter *a*.

In relation to Theorem 3.5, of importance are the following properties of Dickson polynomials. For $n\ge 2$, an *n*-th primitive root of unity $\zeta _n\in \overline{K}, \alpha _k=\zeta _n^k+\zeta _n^{-k}$ and $\beta _k=\zeta _n^k-\zeta _n^{-k}$, we have:

3.2 $$\begin{aligned} \begin{aligned}&D_n(x, a)-D_n(y,a)=(x-y) \prod _{k=1}^{(n-1)/2} (x^2-\alpha _kxy +y^2+\beta _k^2a)\ \text { when } n\hbox { is odd},\\&D_n(x, a)-D_n(y,a)=(x-y)(x+y) \prod _{k=1}^{(n-2)/2} (x^2-\alpha _kxy +y^2+\beta _k^2a)\ \text { when } n\hbox { is even}. \end{aligned} \end{aligned}$$

Furthermore, we have

3.3 $$\begin{aligned} D_{mn}(x, a)=D_m(D_n(x, a), a^n), \quad m, n\in \mathbb {N}. \end{aligned}$$

One may find proofs in Turnwald’s paper [32].

Here are the main ingredients of the proof of Theorem 3.5, as presented by Turnwald [32]. Note that if *f* is decomposable, then $\phi (x, y)=(f(x)-f(y))/(x-y)$ is clearly reducible over *K*. By (3.2), one easily sees that the second statement clearly implies the first. If *f* is of composite degree and $f(x)= e_1 D_n(c_1x+c_0, \alpha )+e_0$ with $e_i, c_i,\alpha \in K$, i.e. *f* is *linearly related to Dickson polynomial*, then *f* is decomposable since (3.3) holds. To prove the converse, assume that *f* is indecomposable. Then ${{\mathrm{Mon}}}(f)$, where *f* is seen as with coefficients in $\overline{K}$, is primitive, by Proposition 3.1 and Proposition 3.2. By Lemma 3.3, $\phi (x, y)$ is ireducible over $\overline{K}$ if and only ${{\mathrm{Mon}}}(f)$, where *f* is seen as with coefficients in $\overline{K}$, is doubly transitive. It remains to show that for *f* over an algebraically closed field, such that ${{\mathrm{Mon}}}(f)$ is primitive but not doubly transitive, it must be that either $f(x)=e_1 D_n(c_1x+c_0, \alpha )+e_0, n>3$ is a prime and $e_i, c_i,\alpha \in K$, or $f(x)=e_1 D_3(c_1x+c_0,0)+e_0=e_1(c_1x+c_0)^3+e_0$ and $e_i, c_i\in K$. In showing that, of key importance is the following lemma.

#### Lemma 3.6

Let *K* be a field with ${{\mathrm{char}}}(K)=0$ and $f\in K[x]$ with $n=\deg f\ge 3$. If ${{\mathrm{Mon}}}(f)$ is primitive, but not doubly transitive, then *n* is prime and for any $y_0\in \overline{K}, f(x)-y_0$ is either an *n*-th power or has one simple root and $(n-1)/r$ roots of multiplicity *r*.

#### Proof

Assume that ${{\mathrm{Mon}}}(f)$ is primitive, but not doubly transitive. Then ${{\mathrm{Mon}}}(f)$ is of prime degree by the above stated Schur’s result. By the above stated Burnside’s result, ${{\mathrm{Mon}}}(f)$ may be identified with a group of affine transformations $ax+b$ of $\mathbb {Z}/n\mathbb {Z}$. If $a=1, b=0$, this permutation is identity, if $a=1, b\ne 0$ it is an *n*-cycle, and if $a\ne 1$, then it is of cycle type $1, r, \ldots , r$, where *r* is the least positive integer such that $a^r=1$. By Lemma 3.4, it follows that for any $y_0\in \overline{K}, f(x)-y_0$ is either a *n*-th power or has one simple root and $(n-1)/r$ roots of multiplicity *r*. $\square$

Fried [12] showed that the only polynomials of degree $n\ge 3$ such that for any $y_0\in \overline{K}, f(x)-y_0$ is either an *n*-th power or has one simple root and $(n-1)/r$ roots of multiplicity *r*, are linearly related to a Dickson polynomial. A proof can be found in [32].

### Polynomials with distinct critical values

In this section as well, *K* is an arbitrary field of characteristic zero.

#### Lemma 3.7

Let *K* be a field with ${{\mathrm{char}}}(K)=0$ and let $f, g, h\in K[x]$ be such that $f(x)=g(h(x))$ and $\deg g>1$. Then for any critical point $\gamma _0\in \overline{K}$ of *g* and $\gamma =g(\gamma _0)$ we have that every root of $h(x)-\gamma _0$ is a root of both $f(x)-\gamma$ and $f'(x)$.

#### Proof

If $h(x_0)=\gamma _0$, then $f(x_0)=g(h(x_0))=g(\gamma _0)=\gamma$ and $f'(x_0)=g'(h(x_0))h'(x_0)=g'(\gamma _0)h'(x_0)=0$. $\square$

Recall that a polynomial is called *Morse* if it has all simple critical points and all distinct critical values. Note that if $f\in K[x]$ is Morse, then *f* is indecomposable by Lemma 3.7. If $f\in K[x]$ has all simple critical points and equal critical values at at most two distinct critical points, by Lemma 3.7 it follows that if $f(x)=g(h(x))$ with $\deg g>1$, then $\deg h\le 2$.

By following the approach of Turnwald [32] and by using Fried’s techniques for proving Theorem 3.5, described in the previous section, we prove the following proposition.

#### Proposition 3.8

Let *K* be a field with ${{\mathrm{char}}}(K)=0$ and let $f\in K[x]$ have at least two distinct critical points and all distinct critical values. Then ${{\mathrm{Mon}}}(f)$ is a doubly transitive permutation group.

#### Proof

We first show that ${{\mathrm{Mon}}}(f)$ is primitive, i.e. that *f* is indecomposable. Assume to the contrary and write $f(x)=g(h(x))$, where $\deg g\ge 2, \deg h\ge 2, h$ is monic and $h(0)=0$ (as in (3.1)). Let $\gamma _0\in \overline{K}$ be a root of $g'$ and $\gamma =g(\gamma _0)$. Then every root of $h(x)-\gamma _0$ is a root of both $f(x)-\gamma$ and $f'(x)$ by Lemma 3.7. If there exist two distinct roots of $h(x)-\gamma _0$, say $x_0$ and $x_1$, then $f'(x_0)=f'(x_1)=0$ and $f(x_0)=f(x_1)=\gamma$, which cannot be by assumption. Thus, $h(x)-\gamma _0$ does not have two distinct roots, i.e. $h(x)=(x-x_0)^k+\gamma _0$, where $k=\deg h\ge 2$. Also, if there exist two distinct roots of $g'$, say $\gamma _0$ and $\gamma _1$, then analogously $h(x)=(x-x_1)^k+\gamma _1$ for some $x_1\in \overline{K}$. Then $(x-x_0)^k+\gamma _0=(x-x_1)^k+\gamma _1$. By taking derivative, we get $k(x-x_0)^{k-1}=k(x-x_1)^{k-1}$, and thus $x_0=x_1$, since $k-1\ge 1$. Then also $\gamma _0=\gamma _1$, a contradiction. Thus $g'(x)=a(x-\gamma _0)^{t-1}$, where $t=\deg g\ge 2$ and $a\in K$. Then

$$\begin{aligned} f'(x)=g'(h(x))h'(x)&=a k(h(x)-\gamma _0)^{t-1}(x-x_0)^{k-1}=\\&=ak (x-x_0)^{k(t-1)}(x-x_0)^{k-1}=ak(x-x_0)^{n-1}. \end{aligned}$$

However, this contradicts the assumption that $f'$ has at least two distinct roots. Thus, ${{\mathrm{Mon}}}(f)$ is primitive.

Assume that ${{\mathrm{Mon}}}(f)$ is not doubly transitive. If $x_0$ is a critical point of *f*, then the multiplicities of the roots of $f(x)-f(x_0)$ are $1, 1, \ldots , 1, k$, where $k\ge 2$ is the multiplicity of $x_0$, since *f* has all distinct critical values. By Lemma 3.6, it follows that $k=p-1$, where $p=\deg f$ (and *p* is prime), so that $x_0$ is a root of $f'$ of multiplicity $p-2$. If $x_1\ne x_0$ is another root of $f'$, then in the same way the multiplicity of $x_1$ is $p-2$. So, $2(p-2)\le p-1$, and $p\le 3$. If $p=3$, then $k=2$, and ${{\mathrm{Mon}}}(f)$ contains an element of cycle type 1, 2 by Lemma 3.4. Since ${{\mathrm{Mon}}}(f)$ is a primitive permutation group and contains a transposition, it is symmetric by Jordan’s theorem [33, Thm. 13.3]. In particular, ${{\mathrm{Mon}}}(f)$ is doubly transitive, a contradiction. $\square$

Let *K* be a field with ${{\mathrm{char}}}(K)=0$ and $f\in K[x]$. Note that if *f* has no two distinct critical points, then $f'(x)=a(x-x_0)^{n-1}$, and thus $f(x)=a/n(x-x_0)^n+\text {const.}$ Such polynomial is indecomposable if and only if *n* is prime. If *f* has two distinct critical points, but has at two equal critical values, then *f* can be decomposable. Indeed, $f(x)=(x^2-1)^2, f'(x)=4x(x^2-1), f(1)=f(-1)=0$. This case will be discussed in more detail in the following section.

#### Corollary 3.9

Let *K* be a field with ${{\mathrm{char}}}(K)=0$ and let $f\in K[x]$ have a critical point of multiplicity at most 2 and all distinct critical values. Then ${{\mathrm{Mon}}}(f)$ is either alternating or symmetric.

#### Proof

Note that for such *f* either $\deg f\in \{2, 3\}$ or *f* has at least two distinct critical points. If the former holds, then *f* is indecomposable, i.e. ${{\mathrm{Mon}}}(f)$ is primitive, since $\deg f$ is prime. In the latter case, ${{\mathrm{Mon}}}(f)$ is primitive by Proposition 3.8. If $x_0$ is a root of $f'$ of multiplicity at most 2, it follows that all the roots of $f(x)-f(x_0)$, but $x_0$, are of multiplicity 1 (since *f* has all distinct critical values), and $x_0$ is of multiplicity $\le 3$. By Lemma 3.4, ${{\mathrm{Mon}}}(f)$ contains either a 2-cycle or a 3-cycle. Since ${{\mathrm{Mon}}}(f)$ is primitive and contains either a 2-cycle or 3-cycle, it is either alternating or symmetric by [33, Thm. 13.3]. $\square$

#### Remark 3.10

Turnwald [32] showed that if $f\in K[x]$ has one simple critical point and all distinct critical values, then ${{\mathrm{Mon}}}(f)$ is symmetric, by the same argument as in the proof of Corollary 3.9. Namely, in this case ${{\mathrm{Mon}}}(f)$ is primitive and contains a 2-cycle, so it is symmetric by [33, Thm. 13.3]. Note that if $f\in K[x]$ is indecomposable and has a simple critical point $x_0$ such that $f(x_1)\ne f(x_0)$ for any other critical point $x_1$ of *f*, then ${{\mathrm{Mon}}}(f)$ is symmetric by the same argument. Also, if $f\in K[x]$ is indecomposable, has at least two distinct critical points, and has a critical point $x_0$ such that $f(x_1)\ne f(x_0)$ for any other critical point $x_1$ of *f*, then ${{\mathrm{Mon}}}(f)$ is doubly transitive by the same argument as in Proposition 3.8. Recall that *f* is indecomposable if $\deg f$ is prime. If $f\in K[x]$ is such that the derivative $f'$ is irreducible over *K*, then *f* is indecomposable over *K* by $f'(x)=g'(h(x))h'(x)$.

#### Remark 3.11

In this paper, we are restricting our attention to the case of fields of characteristic zero. We are doing so for simplicity and since our main results hold over number fields. However, several results hold, under certain assumptions, over fields of positive characteristic. Lemma 3.2 and Lemma 3.3 hold if $f'(x)\ne 0$. Lemma 3.4 holds if ${{\mathrm{char}}}(K)$ does not divide the multiplicites of zeros of $f(x)-x_0\in \overline{K}[x]$. For details, see [32]. By the same proof as in Proposition 3.8, if *K* is a field and $f\in K[x]$ is such that ${{\mathrm{char}}}(K)\not \mid \deg f$ and *f* has at least two distinct critical points and all distinct critical values, then ${{\mathrm{Mon}}}(f)$ is primitive. If *f* has a simple critical point $x_0$ and all distinct critical values, then the multiplicities of the roots of $f(x)-f(x_0)$ are $1, 1, \ldots , 1, 2$. Thus, if ${{\mathrm{char}}}(K)\ne 2$, then ${{\mathrm{Mon}}}(f)$ contains a transposition. If ${{\mathrm{char}}}(K)=2$, then $f''(x)=0$, which is in contradiction with the assumption that $x_0$ is a simple critical point. Thus, if *K* is an arbitrary field and $f\in K[x]$ is such that ${{\mathrm{char}}}(K)\not \mid \deg f$, and *f* has a simple critical point and all distinct critical values, then ${{\mathrm{Mon}}}(f)$ is symmetric. This result and the proof are due to Turnwald [32]. One may prove an analogue of Proposition 3.8 over a field of positive characteristic using the same tools.

Recall that if *K* is a field with ${{\mathrm{char}}}(K)=0$ and $f\in K[x]$, then $(f(x)-f(y))/(x-y)$ is irreducible over *K* if and only if ${{\mathrm{Mon}}}(f)$ is doubly transitive. We remark that questions about reducibility of polynomials of type $(f(x)-f(y))/(x-y)$ have occured in various contexts. For example, a polynomial with coefficients in an integral domain *R* is said to be a *permutation polynomial* modulo an ideal *I* of *R* if the mapping induced on the residue class ring *R* / *I* is bijective. By proving Theorem 3.5, Fried [12] proved the so called *Schur’s conjecture*, which states that every integral polynomial which is a permutation polynomial for infinitely many primes *p* is a composition of linear polynomials and Dickson polynomials. Namely, Fried observed that a polynomial which is a permutation polynomial for infinitely many prime ideals can be written as a composition of indecomposable polynomials *f* such that the polynomial $(f(x)-f(y)/(x-y)$ is not absolutely irreducible.

Several authors have studied functional equations of type $f(a)=g(b)$, where *f*, *g* are given complex polynomials, which are to be solved in non-constant meromorphic complex functions *a*, *b*, and in particular the case when $g(x)=cf(x)$ for some $c\in \mathbb {C}$. Recently, Pakovich [23] studied the case when one allows *f* and *g* to be rational functions. To the study of such questions of importance are questions about reducibility of the curve $f_1(x)g_2(y)-f_2(x)g_1(y)=0$, where $f=f_1/f_2, g=g_1/g_2$, and $f_1, f_2$, as well as $g_1, g_2$, have no common roots (that is the curve obtained by equating to zero the numerator of $f(x)=g(y)$), and reducibility of the curve $(f_1(x)f_2(y)-f_2(x)f_1(y))/(x-y)=0$, where $f_1, f_2\in \mathbb {C}[x]$ have no common roots (corresponding to the case $f=g, c=1$). Namely, by Picard’s theorem from complex analysis there exists a solution of the above functional equation if and only if the corresponding curve has an irreducible component of genus at most one. For a rational function *f* with complex coefficients, Pakovich [23] called $s\in \mathbb {C}$ a critical value of *f* if the set $f^{-1}\{s\}$ contains less than $\deg f$ points. This is compatible with our definition of a critical value of a polynomial. He further called *s* a simple critical value of *f* if $f^{-1}\{s\}$ contains exactly $\deg f-1$ points. In the present paper, these are the critical values at simple critical points. Pakovich [23] showed that if *f* and *g* have at most one common critical value, then the corresponding curve is irreducible. He further showed that the curve $(f_1(x)f_2(y)-f_2(x)f_1(y))/(x-y)=0$, where $f_1, f_2\in \mathbb {C}[x]$ have no common roots, is irreducible if $f=f_1/f_2$ is indecomposable and has at least one simple critical value, or if all critical values of *f* are simple. In the case of polynomials, the former result is contained in Remark 3.10. The latter result is due to Serre [27], and it is generalized by Proposition 3.8.

### Polynomials with at most two equal critical values

In this section, we prove Proposition 1.4. We do that through few lemmas.

#### Lemma 3.12

Let *K* be a field with ${{\mathrm{char}}}(K)=0$ and let $f\in K[x]$ have at least two distinct critical points and equal critical values at at most two distinct critical points.

Assume that $f(x)=g(h(x))$, where $g, h\in K[x]$ and $t=\deg g>1$. Then for any critical point $\gamma _0\in \overline{K}$ of *g*, we have that $h(x)=b(x-x_0)^{k_0}(x-y_0)^{l_0}+\gamma _0$ for some distinct $x_0, y_0\in \overline{K}$, integers $k_0, l_0\ge 0$ such that $k_0+l_0=k=\deg h$, and nonzero $b\in K$. If in addition there do not exist two distinct critical points of *g*, then the derivative of *f* satisfies (1.3).

#### Proof

Let $\gamma _0\in \overline{K}$ be a root of $g'$ and $\gamma =g(\gamma _0)$. Then every root of $h(x)-\gamma _0$ is a root of both $f(x)-\gamma$ and $f'(x)$ by Lemma 3.7. If there exist three distinct roots of $h(x)-\gamma _0$, say $x_0, y_0, z_0$, then $f'(x_0)=f'(y_0)=f'(z_0)=0$ and $f(x_0)=f(y_0)=f(z_0)=\gamma$, which cannot be by assumption. Thus $h(x)-\gamma _0$ does not have three distinct roots, i.e. $h(x)=b(x-x_0)^{k_0}(x-y_0)^{l_0}+\gamma _0$ for some distinct $x_0, y_0\in \overline{K}$, integers $k_0, l_0\ge 0$ such that $k_0+l_0=k=\deg h$, and nonzero $b\in K$. If there do not exist two distinct roots of $g'$, then $g'(x)=a(x-\gamma _0)^{t-1}$, where $t=\deg g\ge 2$ and $a\in K$. Then

$$\begin{aligned} f'(x)&=g'(h(x))h'(x)=a (h(x)-\gamma _0)^{t-1}h'(x)\\&=ab^t (x-x_0)^{k_0(t-1)}(x-y_0)^{l_0(t-1)}(x-x_0)^{k_0-1}(x-y_0)^{l_0-1}(kx -k_0y_0-l_0x_0)\\&=ab^t(x-x_0)^{k_0t-1}(x-y_0)^{l_0t-1}(kx -k_0y_0-l_0x_0). \end{aligned}$$

Moreover, $k_0, l_0\ge 1$ (since otherwise *f* has no two distinct critical points). Thus, (1.3) holds. $\square$

#### Lemma 3.13

Let *K* be a field with ${{\mathrm{char}}}(K)=0$ and let $f\in K[x]$ have at least two distinct critical points and equal critical values at at most two distinct critical points.

Assume that $f(x)=g(h(x))$, where $g, h\in K[x], \deg g>1$ and $\deg h=2$. If *g* has at least two distinct critical points, then ${{\mathrm{Mon}}}(g)$ is doubly transitive.

If *f* is indecomposable and $\deg f\ge 6$, then ${{\mathrm{Mon}}}(f)$ is doubly transitive.

#### Proof

Assume that $f(x)=g(h(x))$, where $g, h\in K[x], \deg g>1, \deg h=2$ and *g* has at least two distinct critical points. Without loss of generality we may assume that *h* is monic and $h(0)=0$, by (3.1). Let $\gamma _0$ and $\gamma _1$ be two distinct roots of $g'$. We now show that $g(\gamma _0)\ne g(\gamma _1)$. Assume to the contrary that $g(\gamma _0)= g(\gamma _1)=\gamma$. Let $x_0$ be a root of $h(x)-\gamma _0$ and $x_1$ a root of $h(x)-\gamma _1$. By Lemma 3.7, it follows that $f'(x_0)=f'(x_1)=0$ and $f(x_0)=g(\gamma _0)=\gamma =g(\gamma _1)=f(x_1)$. Since $\deg h=2$, by assumption it follows that neither $h(x)-\gamma _0$ nor $h(x)-\gamma _1$ have two distinct roots, i.e. $h(x)-\gamma _0=(x-x_0)^2, h(x)-\gamma _1=(x-x_1)^2$. Then $h'(x)=2(x-x_0)=2(x-x_1)$, and thus $x_0=x_1$ and $\gamma _0=\gamma _1$, a contradiction. By Proposition 3.8, it follows that ${{\mathrm{Mon}}}(g)$ is doubly transitive.

Assume that *f* is indecomposable and $\deg f\ge 6$, but ${{\mathrm{Mon}}}(f)$ is not doubly transitive. By Proposition 3.8, there exist two distinct critical points $x_0$ and $x_1$ of *f* such that $f(x_0)=f(x_1)$. The multiplicities of the roots of $f(x)-f(x_0)$ are $1, 1, \ldots , 1,k_0, k_1$, where $k_1\ge 2$ is the multiplicity of $x_0$ and $k_0\ge 1$. Namely, *f* has equal critical values at at most two distinct critical points. By Lemma 3.6, either $k_0=k_1=(p-1)/2$ or $k_0=1, k_1=p-1$, where $p=\deg f$ (and *p* is prime). Note that neither $x_0$ nor $x_1$ cannot be roots of $f'$ of multipicity $p-2$, since $p-2+(p-1)/2-1\le p-1$ only when $p\le 5$. Thus, $f'$ has at most four distinct roots: $x_0, x_1$ are of multiplicity $(p-1)/2-1$, and there is either another double root $x_2$, or two other simple roots $x_2$ and $x_3$. If $x_2$ is a double root of $f'$, then by assumption and Lemma 3.6, the mutiplicities of the roots of $f(x)-f(x_2)$ are 1, 3, so $\deg f=4<6$, a contradiction. Analogously, if $f'$ has simple roots $x_2$ and $x_3$, the mutiplicities of the roots of $f(x)-f(x_2)$ (as well as of $f(x)-f(x_3)$) are either 1, 2 or 1, 2, 2, and in both cases $\deg f<6$, a contradiction. $\square$

#### Proof of Proposition 1.4

Assume that $f(x)=g(h(x))$, where $\deg g>1$ and $\deg h>2$, and without loss of generality assume that *h* is monic and $h(0)=0$ (as in (3.1)). By Lemma 3.12, if there do not exist two distinct roots of $g'$, then (1.3) holds. Assume henceforth that there exist two distinct roots of $g'$, say $\gamma _0$ and $\gamma _1$. By Lemma 3.12 it follows that

3.4 $$\begin{aligned} h(x)=(x-x_0)^{k_0}(x-y_0)^{l_0}+\gamma _0=(x-x_1)^{k_1}(x-y_1)^{l_1}+\gamma _1 \end{aligned}$$

for some distinct $x_0, y_0\in \overline{K}$ such that $k_0, l_0\ge 0$ and $k_0+l_0=k=\deg h$, and distinct $x_1, y_1\in \overline{K}$ such that $k_1, l_1\ge 0$ and $k_1+l_1=k=\deg h$. Assume without loss of generality that $k_0\ge l_0$ and $k_1\ge l_1$. If $l_0=0$ and $l_1=0$, then $h(x)-\gamma _0=(x-x_0)^k$ and $h(x)-\gamma _1=(x-x_1)^k$. Thus, $h'(x)=k(x-x_0)^{k-1}=k(x-x_1)^{k-1}$, and $x_0=x_1$ since $k-1>1$. Then also $\gamma _0=\gamma _1$, a contradiction. If $l_0=0$ and $l_1>0$, then

$$\begin{aligned} h'(x)=k(x-x_0)^{k-1}=(x-x_1)^{k_1-1}(x-y_1)^{l_1-1}(kx -k_1y_1-l_1x_1). \end{aligned}$$

It follows that $kx_0=k_1y_1+l_1x_1$, $l_1=1, k_1=k-1$ and $x_0=x_1$. Then $x_0=y_1$, and hence $x_1=y_1$, a contradiction. We conclude that $k_0, l_0, k_1, l_1\ge 1$, and by taking derivative in (3.4) we get

3.5 $$\begin{aligned} (x-x_0)^{k_0-1}(x-y_0)^{l_0-1}(kx -k_0y_0-l_0x_0)=(x-x_1)^{k_1-1}(x-y_1)^{l_1-1}(kx -k_1y_1-l_1x_1). \end{aligned}$$

If (3.5) holds with $l_0=1$, then $l_1=1$, and vice versa. Namely, otherwise on the left and the right hand side we do not have equal number of distinct roots. If $l_0=l_1=1$, then $k_0=k_1=k-1>1$ and

$$\begin{aligned} (x-x_0)^{k_0-1}(kx -k_0y_0-x_0)=(x-x_1)^{k_0-1}(kx-k_0y_1-x_1). \end{aligned}$$

If $x_0=x_1$, then $k_0y_0 +x_0=k_0y_1+x_0$, so $y_0=y_1$ and hence $\gamma _0=\gamma _1$, a contradiction. Thus, $k_0=k_1=2, k=3$, and $3x_0=x_1+2y_1$ and $3x_1=x_0+2y_0$. Then from (3.4) it follows that $\gamma _1=\gamma _0+x_1^2y_1-x_0^2y_0$. Moreover, $2(\gamma _1-\gamma _0)=(x_0-x_1)^3$ and $3^3(\gamma _1-\gamma _0)=2^2(x_0-y_0)^3$. It follows that there are exactly two distinct roots of $g'$, i.e. $g'(x)=a(x-\gamma _0)^{t_0}(x-\gamma _1)^{t_1}$, where $t_0+t_1=t-1=\deg g-1, a\in K{\setminus } \{0\}$ and $t_0, t_1\ge 1$. Therefore,

$$\begin{aligned} f'(x)= & {} a (h(x)-\gamma _0)^{t_0}(h(x)-\gamma _1)^{t_1}h'(x)\\= & {} 3a (x-x_0)^{2t_0+1}(x-y_0)^{t_0}(x-x_1)^{2t_1+1}(x-y_1)^{t_1}, \end{aligned}$$

and because of $3x_0=x_1+2y_1$ and $3x_1=x_0+2y_0$, it follows that $x_0, y_0, x_1, y_1$ are all distinct. Thus, (1.4) holds.

Assume henceforth that in (3.5) we have $k_0, l_0, k_1, l_1>1$. If $l_0=2$, then $l_1=2$, and vice versa. Namely, otherwise on the left and the right hand side of (3.5) we do not have equal number of simple roots. If $l_0=l_1=2$ and $k_0=k_1>2$, then $x_0=x_1$ and either $y_0=y_1$, or $ky_0 -k_0y_1-2x_0=0, ky_1 -k_0y_0-2x_0=0$. In both cases, $y_0=y_1$, and hence $\gamma _0=\gamma _1$, a contradiction. Assume that $l_0=l_1=k_0=k_1=2$. By Vieta’s formulae and (3.5), it follows that $x_0+y_0=x_1+y_1$ and $x_0y_0=x_1y_1$. Thus, either $x_0=x_1$ and $y_0=y_1$, or $x_0=y_1$ and $y_0=x_1$. In both cases, $\gamma _0=\gamma _1$, a contradiction. If $k_0, l_0, k_1, l_1> 2$, then it follows immediately by (3.5) that either $x_0=x_1$ and $y_0=y_1$, or $x_0=y_1$ and $y_0=x_1$, a contradiction as in the previous case. Lemma 3.12 and Lemma 3.13 complete the proof. $\square$

Note that if (1.3) in Proposition 1.4 holds, then

$$\begin{aligned} f(x)=c_1\left( (x-x_0)^{k_0}(x-y_0)^{l_0}\right) ^{t}+c_0, \end{aligned}$$

for some $c_0, c_1\in K, c_1\ne 0$. If $f(x)=g(h(x))$, where *h* is monic and $h(0)=0$ (which we can assume without loss of generality by (3.1)), then the proof of Proposition 1.4 shows that $g(x)=c_1 (x-\gamma _0)^{t}+c_0, h(x)=(x-x_0)^{k_0}(x-y_0)^{l_0}+\gamma _0$ and $(-1)^{k-1} x_0^{k_0}y_0^{l_0}=\gamma _0$. In this case, *f* clearly has equal critical values at at most two distinct critical points, and $\deg h$ can be arbitrarily large.

If (1.4) holds, and $f(x)=g(h(x))$, where *h* is monic and $h(0)=0$, then the proof of Proposition 1.4 shows that

$$\begin{aligned} h(x)=(x-x_0)^2(x-y_0)+\gamma _0=(x-x_1)^2(x-y_1)+\gamma _1, \quad g'(x)=c_1(x-\gamma _0)^{t_0}(x-\gamma _1)^{t_1}, \end{aligned}$$

for $c_1\ne 0$, and distinct $\gamma _0, \gamma _1\in \overline{K}$ such that $\gamma _0=x_0^2y_0, \gamma _1=x_1^2y_1$. Then also $2(\gamma _1-\gamma _0)=(x_0-x_1)^3$. Clearly, $f(x_0)=f(y_0)$ and $f(x_1)=f(y_1)$, since $h(x_0)=h(y_0)=\gamma _0$ and $h(x_1)=h(y_1)=\gamma _1$. If *f* has equal critical values at more than two distinct critical points, then $g(\gamma _0)=g(\gamma _1)$ and $f(x_0)=f(y_0)=f(x_1)=f(y_1)$. Note that if e.g. $\gamma _0=-1, \gamma _1=1, t_0=t_1=1$, and $c_1=3$, then $g'(x)=3(x^2-1)$, and hence $g(x)=x^3-3x+\text {const.}$ Clearly, $g(-1)\ne g(1)$, so in this case, *f* has equal critical values at at most two distinct critical points and $\deg h=3$.

In relation to the last statement of Proposition 1.4, one can easily check that if $n=5$ and $a\ne 0$, the Dickson polynomial $D_5(x, a)=x^5-5ax^3+5a^2x$ has only simple critical points and equal critical values at at most two distinct critical points, it is indecomposable and its monodromy group is not doubly transitive since (3.2) holds.

## Proofs of the main theorems

### Proof of Theorem 1.1

If the equation $f(x)=g(y)$ has infinitely many solutions with a bounded $\mathcal {O}_S$-denominator, then by Theorem 2.1 we have

4.1 $$\begin{aligned} f(x)=\phi (f_1(\lambda (x))), \quad g(x)=\phi (g_1(\mu (x))), \end{aligned}$$

where $(f_1, g_1)$ is a standard or specific pair over $K, \phi , \lambda , \mu \in K[x]$ and $\deg \lambda =\deg \mu =1$.

By assumption and Proposition 3.8 it follows that ${{\mathrm{Mon}}}(f)$ and ${{\mathrm{Mon}}}(g)$ are primitive permutation groups. Thus *f* and *g* are indecomposable.

Assume that $h:=\deg \phi >1$. Then $\deg f_1=1$ and $\deg g_1=1$, since *f* and *g* are indecomposable. Then from (4.1) it follows that $f(x)=g(\mu (x))$ for some linear $\mu \in K[x]$.

If $\deg \phi =1$, then from (4.1) it follows that

4.2 $$\begin{aligned} f(x)=e_1f_1(c_1x+c_0)+e_0,\quad g(x)=e_1g_1(d_1x+d_0)+e_0, \end{aligned}$$

for some $c_1,c_0, d_1, d_0, e_1, e_0\in K$, and $c_1d_1e_1\ne 0$. Let $\deg f=\deg f_1=:k$ and $\deg g=\deg g_1=:l$. By assumption $k, l\ge 3$.

Note that $(f_1, g_1)$ cannot be a standard pair of the second kind, since $k, l\ge 3$.

If $(f_1, g_1)$ is a standard pair of the fifth kind, then either $f_1(x)=(ax^2-1)^3$ or $g_1(x)=(ax^2-1)^3$. By (4.2) it follows that either *f* or *g* are decomposable, a contradiction.

If $(f_1, g_1)$ is a standard pair of the first kind, then either $f_1(x)=x^{k}$ or $g_1(x)=x^{l}$. Since $f'$ and $g'$ have at least two distinct roots, we have a contradiction with (4.2).

If $(f_1, g_1)$ is a standard pair of the third or of the fourth kind, then

4.3 $$\begin{aligned} f(x)=e_2D_{k}(c_1x+c_0, a)+e_0,\quad g(x)=e_2'D_{l}(d_1x+d_0, b)+e_0, \end{aligned}$$

where $\gcd (k, l)\le 2$ and $e_2, e_2', a, b\in K{\setminus }\{0\}$. However, this cannot be. Namely, since $k, l\ge 3$ and $\gcd (k, l)\le 2$, it follows that either $k\ge 4$ or $l\ge 4$. Assume $k\ge 4$. By Proposition 3.8, it follows that also when we consider *f* as with coefficients in $\overline{K}$, the monodromy group of *f* over $\overline{K}$ is doubly transitive. Then $(f(x)-f(y))/(x-y)$ is irreducible over $\overline{K}$ by Lemma 3.3. This is in contradiction with (3.2). We conclude analogously if $l\ge 4$.

If $(f_1, g_1)$ is a specific pair, then

4.4 $$\begin{aligned} f(x)=e_2D_{k}(\gamma _1x+\gamma _0, a)+e_0,\quad g(x)=-e_2D_{l}(\delta _1x+\delta _0, b)+e_0, \end{aligned}$$

for some $\gamma _1, \delta _1, \gamma _0, \delta _0\in \overline{K}, e_2, a, b\in K{\setminus }\{0\}$, where $\gcd (k, l)\ge 3$. This, by the same argument as above, cannot be unless $(k, l)=(3, 3)$. If $(k, l)=(3, 3)$, then either $f_1(x)=D_3(x, a)=x^3-3xa$ and $g_1(x)=-D_3(1/2x, a)=-1/8x^3+3/2xa$, or vice versa. Thus, either $g_1(-2x)=f_1(x)$ or $f_1(-2x)=g_1(x)$. From (4.2) it follows that $g(\mu (x))=f(x)$ for some linear $\mu \in K[x]$. $\square$

To the proof of Theorem 1.5 we need the following lemma.

### Lemma 4.1

Let *K* be a field with ${{\mathrm{char}}}(K)=0$ and let $f\in K[x]$ have at least two distinct critical points and equal critical values at at most two distinct critical points. Assume that $f(x)= e_1D_n(c_1x+c_0, a)+e_0$ for some $e_i, c_i, a\in K$ such that $e_1 c_1 a\ne 0$. Then $n\le 5$. Furthermore, the derivative of *f* does not satisfy (1.4), and it satisfies (1.3) only when $n=4$.

### Proof

Since Dickson polynomials have only simple critical points (see e.g. [6, p. 216]), it follows that (1.4) does not hold, and (1.3) can hold only if $k_0=k_1=1$ and $t=2$, so if $n=4$. Since $D_4(x, a)=x^4-4x^2a+2a^2$ and $D_4'(x, a)=4x^3-8a x=4x(x^2-2a)$, it follows that the derivative of $D_4(x, a)$ satisfies (1.3) (with $t=2, k_0=k_1=1, a'=2, x_0=\sqrt{2a}, y_0=-\sqrt{2a}$).

If $n\ge 6$ and *f* is indecomposable, then ${{\mathrm{Mon}}}(f)$ is doubly transitive by Proposition 1.4, which contradicts (3.2). By Proposition 1.4, it follows that it must be that $f(x)=(e_1D_{n/2}(c_1x+c_0, a^2)+e_0)\circ D_2(c_1x+c_0, a)$, where the monodromy group of $(e_1D_{n/2}(c_1x+c_0, a^2)+e_0)$ is doubly transitive. If $n/2\ge 4$, this contradicts (3.2). Thus $n=6$, but then $f(x)= (e_1D_2(c_1x+c_0, a^3)+e_0)\circ D_3(c_1x+c_0, a)$ by (3.3), which contradicts Proposition 1.4 since $\deg D_3(c_1x+c_0, a)=3>2$. $\square$

### Theorem 4.2

Let *K* be a number field, *S* a finite set of places of *K* that contains all Archimedean places, $\mathcal {O}_S$ the ring of *S*-integers of *K* and $f,g\in K[x]$ such that $\deg f\ge 3, \deg g\ge 3$ and $\deg f< \deg g$.

Assume that *f* and *g* both have at least two distinct critical points and equal critical values at at most two distinct critical points, and their derivatives do not satisfy (1.3) nor (1.4). Then the equation $f(x)=g(y)$ has only finitely many solutions with a bounded $\mathcal {O}_S$-denominator, unless either *f* is indecomposable and $g(x)=f(\nu (x))$ for some quadratic $\nu \in K[x]$, or

4.5 $$\begin{aligned} f(x)=e_1D_3(c_1x+c_0, a^5)+e_0, \quad g(x)=e_1D_5(d_1x+d_0, a^3)+e_0, \end{aligned}$$

where $D_n(x, a)$ is the *n*-th Dickson polynomial with parameter $a, c_i, d_i, e_i, a\in K$ and $c_1d_1e_1a\ne 0$.

If both *f* and *g* have only simple critical points and equal critical values at at most two distinct critical points, then the equation $f(x)=g(y)$ has only finitely many solutions with a bounded $\mathcal {O}_S$-denominator, unless either *f* is indecomposable and $g(x)=f(\nu (x))$ for some quadratic $\nu \in K[x]$, or

4.6 $$\begin{aligned} f(x)=e_1f_1(c_1x+c_0)+e_0, \quad g(x)=e_1g_1(d_1x+d_0)+e_0, \end{aligned}$$

where $$ (f_1, g_1)\in \left\{ (D_3(x, a^4), D_4(x, a^3)), (D_3(x, a^5), D_5(x, a^3)), (D_4(x, a^5), D_5(x, a^4))\right\} , c_i, d_i, e_i, a\in K$$ and $$c_1d_1e_1a\ne 0$$.

### Proof

If the equation $f(x)=g(y)$ has infinitely many solutions with a bounded $\mathcal {O}_S$-denominator, then by Theorem 2.1 we have that

4.7 $$\begin{aligned} f(x)=\phi (f_1(\lambda (x))), \quad g(x)=\phi (g_1(\mu (x))), \end{aligned}$$

where $(f_1, g_1)$ is a standard or specific pair over $K, \phi , \lambda , \mu \in K[x]$ and $\deg \lambda =\deg \mu =1$.

Assume that $\deg \phi >1$. Since *f* and *g* are such that their derivatives do not satisfy (1.3) nor (1.4), by Proposition 1.4 if follows that $\deg f_1\le 2$ and $\deg g_1\le 2$. Since $\deg f< \deg g$, it follows that $\deg f_1=1$ and $\deg g_1=2$. Since $\deg g_1=2$, by Proposition 1.4 it further follows that $\phi$ is indecomposable. By (4.7), it follows that $f(\nu _1(x))=\phi (x)$ for some linear $\nu _1\in K[x]$. Then $g(x)=f(\nu (x))$ for some $\nu \in K[x]$ with $\deg \nu =2$.

Assume further that $\deg \phi =1$. Then from (4.7) it follows that

4.8 $$\begin{aligned} f(x)=e_1f_1(c_1x+c_0)+e_0,\quad g(x)=e_1g_1(d_1x+d_0)+e_0, \end{aligned}$$

where $c_1,c_0, d_1, d_0, e_1, e_0\in K$, and $c_1d_1e_1\ne 0$. Let $\deg f=\deg f_1=:k$ and $\deg g=\deg g_1=:l$. By assumption $l>k\ge 3$. Note that since *f* and *g* both have at least two distinct critical points and equal critical values at at most two distinct critical points, by (4.8) the same holds for $f_1$ and $g_1$. Analogously, the derivatives of $f_1$ and $g_1$ do not satisfy (1.3) nor (1.4).

Note that $(f_1, g_1)$ cannot be a standard pair of the second kind, since $k, l>2$.

If $(f_1, g_1)$ is a standard pair of the fifth kind, then $g_1(x)=(ax^2-1)^3$ and $f_1(x)=3x^4-4x^3$. Note that $g_1'(x)=6ax(ax^2-1)^2$, so the derivative of $g_1$ satisfies (1.3) (with $t=3, k_0=k_1=1, a'=3a^3, x_0=1/\sqrt{a}, y_0=-1/\sqrt{a}$), a contradiction.

If $(f_1, g_1)$ is a standard pair of the first kind, then either $f_1(x)=x^{k}$ or $g_1(x)=x^{l}$, so either $f_1$ or $g_1$ do not have two distinct critical points, a contradiction.

If $(f_1, g_1)$ is a standard pair of the third or fourth kind, then $f_1(x)=e_2D_k(x, a)$ and $g_1(x)=e_2'D_l(x, b)$, where $\gcd (k, l)\le 2$ and $e_2, e_2', a, b\in K{\setminus }\{0\}$. By assumption and by Lemma 4.1, it follows that $3\le k<l\le 5$ and $k, l\ne 4$. Thus, $(k, l)=(3, 5)$. Moreover, $e_2=e_2'=1, f_1(x)=D_3(x, a^5), g_1(x)=D_5(x, a^3)$. Thus, (4.5) holds.

If $(f_1, g_1)$ is a specific pair, then $f_1(x)=e_2D_k(\gamma _1x, a), g_1(x)=-e_2D_l(\gamma _2x, b)$ for some $\gamma _1, \gamma _2\in \overline{K}$ with $\gcd (k, l)\ge 3, e_2, a, b\in K{\setminus }\{0\}$. By assumption and by Lemma 4.1 it must be that $k, l\le 5, \gcd (k, l)=3$ and $k<l$, a contradiction.

If both *f* and *g* have only simple critical points and equal critical values at at most two distinct critical points, it follows by Proposition 1.4 that their derivatives do not satisfy (1.3) nor (1.4), unless $k_0=k_0=1, t=2$ in (1.3). Thus, the proof of the second statement follows from the proof of the first, except that in the analysis of standard pairs of the third and fourth kind in the case when $\deg \phi =1$, we can not exclude the cases when $k=4$ or $l=4$, since $D_4(x, a)$ is such that its derivative satisfies (1.3) with $k_0=k_0=1, t=2$, but it has only simple critical points and equal critical values at at most two distinct critical points. Thus, if $\deg \phi >1$, we have that *f* is indecomposable and $g(x)=f(\nu (x))$ for some quadratic $\nu \in K[x]$. If $\deg \phi =1$, by (4.8) it follows that (4.6) holds. $\square$

We did not cover the case $\deg f=\deg g$ in Theorem 4.2, as it is somewhat harder to handle. Namely, in the proof of Theorem 4.2 we used that $\deg f<\deg g$ when we concluded that if $f(x)=\phi (f_1(\lambda (x)))$ and $g(x)=\phi (g_1(\mu (x)))$ with $\deg \phi >1$, then $\deg f_1=1$ and $\deg g_1=2$ by Proposition 1.4. If we had allowed $\deg f=\deg g$, then we would have had two more cases to consider, namely the case $\deg f_1=1$ and $\deg g_1=1$, which is easy to handle, and the case $\deg f_1=2$ and $\deg g_1=2$. In the latter case, we couldn’t express easily the relation between *f* and *g*. We remark that in the results in the literature, which we will derive as corollaries of Theorem 4.2 in Sect. 5, in almost all cases it is assumed that $\deg f\ne \deg g$.

## Corollaries of the main theorems

We now present several corollaries of Theorems 1.1 and 1.5. Most of these corollaries are either results of published papers or inspired by those results. We remark that our proofs of Theorems 1.1 and 1.5 are shorter than the proofs in those papers. We first list some corollaries of Theorem 1.1.

As we have seen in the introduction, Theorem 1.1 implies immediately Corollary 1.2, which generalizes the main of result of Péter, Pintér and Schinzel [24, Thm. 1]. They proved it using other tools: Hajós lemma on the multiplicites of roots of lacunary polynomials (see [26, p. 187]), a result of Fried and Schinzel [14] about indecomposability of polynomials in (1.1), and by comparison of coefficients. Corollary 1.3 was shown by Davenport, Lewis and Schinzel [8], by a finiteness criterion developed by them, which is weaker than the later one of Bilu and Tichy [5].

For positive integers $k \le n-1$, let

5.1 $$\begin{aligned} P_{n, k}(x):=\sum _{j=0}^k {n \atopwithdelims ()j} x^j={n \atopwithdelims ()0}+{n \atopwithdelims ()1}x + {n \atopwithdelims ()2} x^2+\cdots +{n \atopwithdelims ()k} x^{k}. \end{aligned}$$

The polynomial $P_{n, k}$ is said to be a *truncated binomial polynomial* at the *k*-th stage.

### Corollary 5.1

Let $n, k, m, l \in \mathbb {N}$ be such that $3\le k\le n-1, 3\le l\le m-1$ and $k\ne l$. If both $P_{n-1,k-1}$ and $P_{m-1,l-1}$ are such that they have no two distinct roots whose quotient is a *k*-th, respectively *l*-th, root of unity, then the equation $P_{n,k}(x)=P_{m,l}(y)$ has only finitely many integral solutions *x*, *y*.

### Proof

It is easy to check that the following two identities hold:

5.2 $$\begin{aligned} \begin{aligned} P_{n, k}'(x)&=nP_{n-1, k-1}(x)\\ P_{n, k}(x)-(x+1)\frac{P_{n, k}'(x)}{n}&={n-1 \atopwithdelims ()k}x^k. \end{aligned} \end{aligned}$$

Clearly, $P_{n, k}$ has all distinct critical points and all distinct critical values, unless it has two critical points whose quotient is a *k*-th root of unity. Thus, the statement follows by Theorem 1.1. $\square$

In [9], Dubickas and Kreso studied the equation $P_{n,k}(x)=P_{m, l}(y)$ from Corollary 5.1. They showed that this equation has only finitely many integral solutions when $2\le k\le n-1, 2\le l\le m-1$, and $k\ne l$, by assuming irreducibility of $P_{n-1, k-1}$ and $P_{m-1, l-1}$. Irreducibility of truncated binomial expansions has been studied by several authors and the results suggest that $P_{n,k}$ is irreducible for all $k<n-1$. The existence of two distinct roots of $P_{n-1,k-1}$ whose quotient is a *k*-th root of unity is an open problem when $k<n-1$. Computations show that for $n\le 100$ and $k<n-1$ no such two roots exist. The case $k=n-1$ is solved in [9]. We will discuss this case later, when we will list some corollaries of Theorem 4.2.

### Corollary 5.2

For $m>n\ge 3$, the equation

5.3 $$\begin{aligned} \frac{x^n}{n!}+\cdots +\frac{x^2}{2!}+x+1=\frac{y^m}{m!}+\cdots +\frac{y^2}{2!}+y+1, \end{aligned}$$

has only finitely many integral solutions *x*, *y*.

Kulkarni and Sury [20] proved Corollary 5.2. If *f* is the polynomial on the left hand side of (5.3), then $f(x)=f'(x)+x^n/n!$, and *f* thus has only simple critical points. To see that *f* has all distinct critical values it suffices to show that no two roots of $f'$ are such that their quotient is an *n*-th root of unity. It is shown in [20] that this holds by using the fact that the Galois groups of *f* and $f'$ are either symmetric or alternating, which is a well-known result of Schur.

Note that Theorem 1.1 applies to equations of type $f(x)=g(y)$, where *f* and *g* are any of the above mentioned polynomials. In particular, the following clearly holds.

### Corollary 5.3

For $m, n\ge 3$ and $m\ne n$, the equation

$$\begin{aligned} x^{n}+x^{n-1}+\cdots +x+1=\frac{y^m}{m!}+\cdots +\frac{y^2}{2!}+y+1, \end{aligned}$$

has only finitely many integral solutions.

We now derive some corollaries of Theorem 1.5. To get complete statements of some of the results in the literature, we still need to examine the exceptional cases in Theorem 4.2: If $\deg f<\deg g$ we need to examine the cases when $(\deg f, \deg g)\in \{(3, 4), (3, 5), (4, 5)\}$, and in the case when *f* is indecomposable, we need to examine if $g(x)=f(\nu (x))$. All these cases are easy to handle. In handling the latter case, the following result is useful.

### Proposition 5.4

Let *K* be a field with ${{\mathrm{char}}}(K)=0$. Assume that $f=g_1\circ g_2=h_1\circ h_2$ for some $f, g_1, g_2, h_1, h_2\in K[x]$ such that $\deg g_1 = \deg h_1$, and hence $\deg g_2=\deg h_2$. Then there exists a linear polynomial $\ell \in K[x]$ such that $g_1=h_1\circ \ell$ and $g_2=\ell ^{\langle -1\rangle } \circ h_2$.

A proof of Proposition 5.4 can be found in [36, Cor. 2.9]. The case $K=\mathbb {C}$ was proved by Ritt [25].

The following two results can be found in [9]. We recall the proof of the first one and give a short proof of the second one using Theorem 4.2.

### Lemma 5.5

For positive integer $n\ge 3$, the polynomial $(1+x)^n-x^n$ has at least two distinct critical points and equal critical values at at most two distinct critical points.

### Proof

Note that $(1+x)^n-x^n=P_{n, n-1}(x)$ by (5.1). Assume that there exist two distinct roots $\alpha$ and $\beta$ of $P_{n, n-1}'(x)=n((x+1)^{n-1}-x^{n-1})$ such that $P_{n, n-1}(\alpha )=P_{n, n-1}(\beta )$. Then $(\alpha +1)^{n-1}=\alpha ^{n-1}$, $(\beta +1)^{n-1}=\beta ^{n-1}$ and $\alpha ^{n-1}=\beta ^{n-1}$. Note that the roots of $(x+1)^{n-1}-x^{n-1}$ lie on the vertical line $\mathfrak {R}(z)=-1/2$. Then from $\alpha ^{n-1}=\beta ^{n-1}$ it follows that $\alpha$ and $\beta$ are complex conjugates, since they are distinct but have equal absolute values. $\square$

### Theorem 5.6

For $m>n\ge 3$, the equation

$$\begin{aligned} (1+x)^n-x^n=(1+y)^m-y^m, \end{aligned}$$

has only finitely many integral solutions *x*, *y*.

### Proof

Recall that $(1+x)^n-x^n=P_{n, n-1}(x)$ has only simple critical points by (5.1) and (5.2). By Theorem 4.2 and Lemma 5.5 it follows that the equation has only finitely many integral solutions, unless either $(n, m)\in \{(3, 4), (3, 5), (4, 5)\}$, or $(1+x)^n-x^n$ is indecomposable and $(1+x)^m-x^m=((1+x)^n-x^n)\circ \nu (x)$ for some quadratic $\nu \in K[x]$. We first show that the latter cannot hold. One easily verifies that for $m=2m'+1$ we have

$$\begin{aligned} (1+x)^m-x^m={\tilde{P}}_{m, m-1}(x)\circ (x^2+x), \quad {\tilde{P}}_{m, m-1}(x):= \prod _{j=1}^{m'}\left( (2-\omega _j-\overline{\omega _j})x+1\right) , \end{aligned}$$

where $\omega _j=\exp (2\pi ij/n), j=1, 2, \dots , n$. By Lemma 5.5 and Proposition 1.4, ${\tilde{P}}_{m, m-1}$ is indecomposable for all odd $m>2$, and if $m>2$ is even, then $(1+x)^m-x^m$ is indecomposable. If

$$\begin{aligned} {\tilde{P}}_{m, m-1}(x)\circ (x^2+x)=(1+x)^m-x^m=((1+x)^n-x^n)\circ \nu (x) \end{aligned}$$

for some quadratic $\nu$, then $(1+x)^n-x^n={\tilde{P}}_{m, m-1}(x) \circ \mu _1(x)$ for some linear $\mu _1 \in \mathbb {Q}[x]$ by Proposition 5.4. This cannot be since all the roots of ${\tilde{P}}_{m, m-1}(x)$ are clearly real and the roots of $(1+x)^n-x^n$ are, except for at most one (which is $-1/2$ when *n* is even), clearly all complex. To complete the proof using Theorem 4.2, one has to show that it cannot be that

5.4 $$\begin{aligned} (1+x)^n-x^n=e_1f_1(c_1x+c_0)+e_0, \quad (1+x)^m-x^m=e_1g_1(d_1x+d_0)+e_0, \end{aligned}$$

where $(f_1, g_1)\in \left\{ (D_3(x, a^4), D_4(x, a^3)), (D_3(x, a^5), D_5(x, a^3)), (D_4(x, a^5), D_5(x, a^4))\right\} , c_i, d_i, e_i, a\in K$ and $c_1d_1e_1a\ne 0$. Assume to the contrary that (5.4) holds. By taking derivatives we get

$$\begin{aligned} n((1+x)^{n-1}-x^{n-1})=e_1c_1f_1'(c_1x+c_0), \quad m((1+x)^{m-1}-x^{m-1})=e_1d_1g_1'(d_1x+d_0). \end{aligned}$$

The roots of the polynomials on the left hand side are, except for at most one, all complex, while one immediately sees by (2.2) that when $f_1(x)=D_3(x, a^4)$ or $g_1(x)=D_5(x, a^4)$, all the roots of the polynomials on the right hand side are real (since $a^4\ge 0$ for all $a\in \mathbb {Q}$). Finally, $(1+x)^6-x^6=e_1D_5(d_1x+d_0, a^3)+e_0$ does not hold for any real $d_i, e_i, a\in K$ with $d_1e_1a\ne 0$ by direct comparison of coefficients using (2.1). $\square$

In the sequel we list some families of polynomials that have at least two distinct critical points and equal critical values at at most two distinct critical points. For brevity, we do not recall proofs if there are proofs in the literature. We further list several results from the literature, which can be derived as corollaries of Theorem 4.2.

Stoll [28] showed that if $(y_n)_n$ is a sequence of polynomials with real coefficients that satisfy a differential equation

5.5 $$\begin{aligned} \begin{aligned}&\sigma (x)y_n''(x)+\tau y_n'(x)-\lambda _n y_n(x)=0, \quad n\ge 0, \ \text {where}\\&\sigma , \tau \in \mathbb {R}[x], \ \deg \sigma \le 2, \ \deg \tau \le 1, \ \lambda _n\in \mathbb {R}{\setminus } \{0\}, \ \sigma '-2\tau \not \equiv 0, \end{aligned} \end{aligned}$$

then for all $n\ge 3, y_n$ has equal critical values at at most two distinct critical points. Stoll used this to find the possible decompositions of some classical orthogonal polynomials, namely Hermite, Laguerre, Jacobi, Gegenbauer and Bessel polynomials. They satisfy a differential equation of type (5.5). These polynomials also have all simple real zeros, and thus also all simple critical points, by Rolle’s theorem. (Thus, already by Lemma 3.7 it follows that if $y_n(x)=g(h(x))$, where $g, h\in \mathbb {R}[x]$ and $\deg g>1$, then $\deg h\le 2$). Stoll and Tichy studied Diophantine equations with orthogonal polynomials in [29–31].

### Theorem 5.7

For $m> n\ge 3$ and $d_1, d_2\in \mathbb {Q}$, the equation

$$\begin{aligned} x(x+d_1)\cdots (x+(m-1)d_1)=y(y+d_2)\cdots (y+(n-1)d_2) \end{aligned}$$

has only finitely many integral solutions *x*, *y*.

Theorem 5.7 was proved by Beukers, Shorey and Tijdeman [2]. They also proved that for nonzero $d\in \mathbb {Q}$, and $m\ge 3$, the polynomial $x(x+d)\cdots (x+(m-1)d)$ has at least two distinct critical points and equal critical values at at most two distinct critical points, as a step in finding the possible decompositons of this polynomial. Thus, Theorem 5.7 follows, to the most part, by Theorem 4.2.

### Theorem 5.8

Let $G_0(x)=0, G_1(x)=1$, and for nonzero integer *B* let $G_{n+1}(x)=xG_n(x)+BG_{n-1}(x)$ for $n\in \mathbb {N}$. For $m> n\ge 3$, the equation $G_m(x)=G_n(y)$ has only finitely many integral solutions *x*, *y*.

Theorem 5.8 is due to Dujella and Tichy [10]. It is easy to check, and it was observed by Dujella and Tichy, that $G_n(x)=\mu _1(U_{n-1}(\mu _2(x)))$, where $\mu _1, \mu _2\in K[x]$ are linear polynomials and $U_n$ is the *n*-th Chebyshew polynomial of the second kind, given by a differential equation $(1-x^2)U_n''(x)-3xU_n'(x)'+n(n+2)U_n(x)=0$. One easily finds that $U_{n}$ has simple real roots (since $U_{n}(\cos x)=\sin (n+1)x / \sin x)$, and thus simple critical points as well by Rolle’s theorem. By Stoll’s results on polynomials satisfying differential equation (5.5), it immediately follows that $U_{n}$ has equal critical values at at most two distinct critical points. Dujella and Tichy showed this using a different approach. Thus, Theorem 5.8 follows, to the most part, by Theorem 4.2.

It seems likely that the Bernoulli and Euler polynomials satisfy the conditions of Theorem 4.2. Find more about these polynomials in e.g. [3, 17]. It is well-known that the *k*-th power sum of the first $n-1$ positive integers $S_k(n)=1^k+2^k+\cdots +(n-1)^k$ and the alternating *k*-th power sum of the first $n-1$ positive integers $T_k(n)=-1^k+2^k+\cdots +(-1)^{n-1}(n-1)^k$ can be expressed in terms of Bernoulli polynomial $B_k(x)$ and Euler polynomials $E_k(x)$, as

$$\begin{aligned} S_k(n)=\frac{1}{k+1}\left( B_{k+1}(n)-B_{k+1}\right) , \quad T_k(n)=\frac{1}{2}\left( E_k(0)+(-1)^{n-1} E_k(n)\right) . \end{aligned}$$

In various papers, of which we mention [1, 3, 17], equations of type $\mu _1(B_k(\mu _2(x)))=\lambda _1(B_n(\lambda _2(x)))$, and $\mu _1(E_k(\mu _2(x)))=\lambda _1(E_n(\lambda _2(x)))$, where $\mu _i, \lambda _i\in \mathbb {Q}[x]$ are linear and $k, n\ge 3$, have been studied, corresponding to equations with the above introduced power sums. We do not have a proof at hand, but if Bernoulli and Euler polynomials are such that they have equal critical values at at most two distinct critical points, then Theorem 4.2 would yield a unifying proof of the results in these papers. It is well known that Bernoulli polynomials have simple roots and that $B_n'(x)=nB_{n-1}(x)$, so that they have all simple critical points as well. Also, $E_n'(x)=nE_{n-1}(x)$ and the only Euler polynomial with a multiple root is of degree 5 and has one simple root and two double roots. Finally, note that if Bernoulli and Euler polynomials are such that at least they have equal critical values at at most two distinct critical points, then Theorem 4.2 would also apply to equations of type $\mu _1(B_k(\mu _2(x)))=\lambda _1(E_n(\lambda _2(x)))$ with linear $\mu _i, \lambda _i\in \mathbb {Q}[x]$.
